# Pickaxe: a Python library for the prediction of novel metabolic reactions

**DOI:** 10.1186/s12859-023-05149-8

**Published:** 2023-03-22

**Authors:** Kevin M. Shebek, Jonathan Strutz, Linda J. Broadbelt, Keith E. J. Tyo

**Affiliations:** 1 Department of Chemical and Biological Engineering, Northwestern University, Evanston, IL 60208 USA; 2 Center for Synthetic Biology, Northwestern University, Evanston, IL 60208 USA; 3 Chemistry of Life Processes Institute, Northwestern University, Evanston, IL 60208 USA

**Keywords:** Enzyme promiscuity, Network generation, Biosynthetic design, Retrobiosynthesis, Metabolite identification

## Abstract

**Background:**

Biochemical reaction prediction tools leverage enzymatic promiscuity rules to generate reaction networks containing novel compounds and reactions. The resulting reaction networks can be used for multiple applications such as designing novel biosynthetic pathways and annotating untargeted metabolomics data. It is vital for these tools to provide a robust, user-friendly method to generate networks for a given application. However, existing tools lack the flexibility to easily generate networks that are tailor-fit for a user’s application due to lack of exhaustive reaction rules, restriction to pre-computed networks, and difficulty in using the software due to lack of documentation.

**Results:**

Here we present Pickaxe, an open-source, flexible software that provides a user-friendly method to generate novel reaction networks. This software iteratively applies reaction rules to a set of metabolites to generate novel reactions. Users can select rules from the prepackaged JN1224min ruleset, derived from MetaCyc, or define their own custom rules. Additionally, filters are provided which allow for the pruning of a network on-the-fly based on compound and reaction properties. The filters include chemical similarity to target molecules, metabolomics, thermodynamics, and reaction feasibility filters. Example applications are given to highlight the capabilities of Pickaxe: the expansion of common biological databases with novel reactions, the generation of industrially useful chemicals from a yeast metabolome database, and the annotation of untargeted metabolomics peaks from an *E. coli* dataset.

**Conclusion:**

Pickaxe predicts novel metabolic reactions and compounds, which can be used for a variety of applications. This software is open-source and available as part of the MINE Database python package (https://pypi.org/project/minedatabase/) or on GitHub (https://github.com/tyo-nu/MINE-Database). Documentation and examples can be found on Read the Docs (https://mine-database.readthedocs.io/en/latest/). Through its documentation, pre-packaged features, and customizable nature, Pickaxe allows users to generate novel reaction networks tailored to their application.

**Supplementary Information:**

The online version contains supplementary material available at 10.1186/s12859-023-05149-8.

## Background

While many enzymes are highly optimized to act on specific molecules, it is possible for enzymes to display substrate promiscuity and catalyze a transformation on multiple different substrates [1]. Promiscuity provides the ability to generate unexplored reactions that are not found in existing biological databases and genome scale models. By engineering pathways that utilize promiscuity, novel pathways have been created to generate synthetic carbon fixation cycles, improve pathway performance, and enable target production of non-native products in organisms [2–6]. Promiscuous enzymes have also been used in engineered pathways to produce new-to-nature products [7, 8]. However, promiscuity may have unintended consequences of byproduct formulation leading to low yield of a desired product or the production of chemicals that may be toxic to the cell [9, 10]. Although there have been successes, in order to fully utilize the potential of promiscuity for determining the metabolism of an organism as well as designing novel pathways, it is paramount to be able to understand and enumerate the possible reactions stemming from promiscuous enzymes [11, 12].

Promiscuous reaction generation tools have been developed that utilize substrate promiscuity to generate metabolic networks consisting of novel reactions and compounds [13–19]. These tools rely on reaction rules, which are generated by capturing the underlying transformations and allow for varying degrees of substrate specificity [20]. Applying these rules iteratively to a set of metabolites produces a network consisting of novel reactions that can be used for a variety of applications such as identifying unannotated metabolomics peaks or designing biosynthetic pathways [19, 21–24].

While tools exist that utilize network generation for pathway generation and metabolic applications, these are often limited in the analyses they can accomplish. Ideally, a tool would afford the user control over the reaction rules used, provide the ability to run the software locally, have flexible data outputs, and offer the ability to easily write custom functionality into the code. However, obtaining all these features in a single existing tool is difficult, due to the fact that some work only with a web interface, use pre-computed networks, or are unable to accept user-defined rules [13, 17, 25–28]. Additionally, lack of documentation for open-source tools makes it difficult to develop and work with existing code bases.

Here we present Pickaxe, a flexible biochemical reaction generation tool to generate custom network expansions. This software enables users the ability to tailor reaction prediction through the control of reactions used and the application of filters that allow for on-the-fly pruning of a network. Reaction rules can either be defined by a user, or the prepackaged set of highly comprehensive, yet minimal, reaction rules known as JN1224min generated from MetaCyc can be used [20]. When compared to other open-source reaction rules, the JN1224min ruleset maps the largest percentage of KEGG and BRENDA reactions, providing the most comprehensive coverage of any ruleset. Pickaxe comes with default filters as well as a template that enables users to define their own custom filters. Generated networks can be used directly in a python workflow, written to a comma separated value (CSV) file, or stored in a MongoDB database for further network analysis. Pickaxe runs standalone, but is included within the MINE Database software, which utilizes Pickaxe to generate a database in order to identify potential structures for unannotated metabolomics peaks [29]. This software is open-source and can be found at https://github.com/tyo-nu/MINE-Database or can be installed as a python package (https://pypi.org/project/minedatabase/2.0.0/). Documentation detailing the code and providing examples of Pickaxe applications can be found at https://mine-database.readthedocs.io/en/latest/pickaxe_run.html.

## Implementation

### Overview

**Fig. 1:**
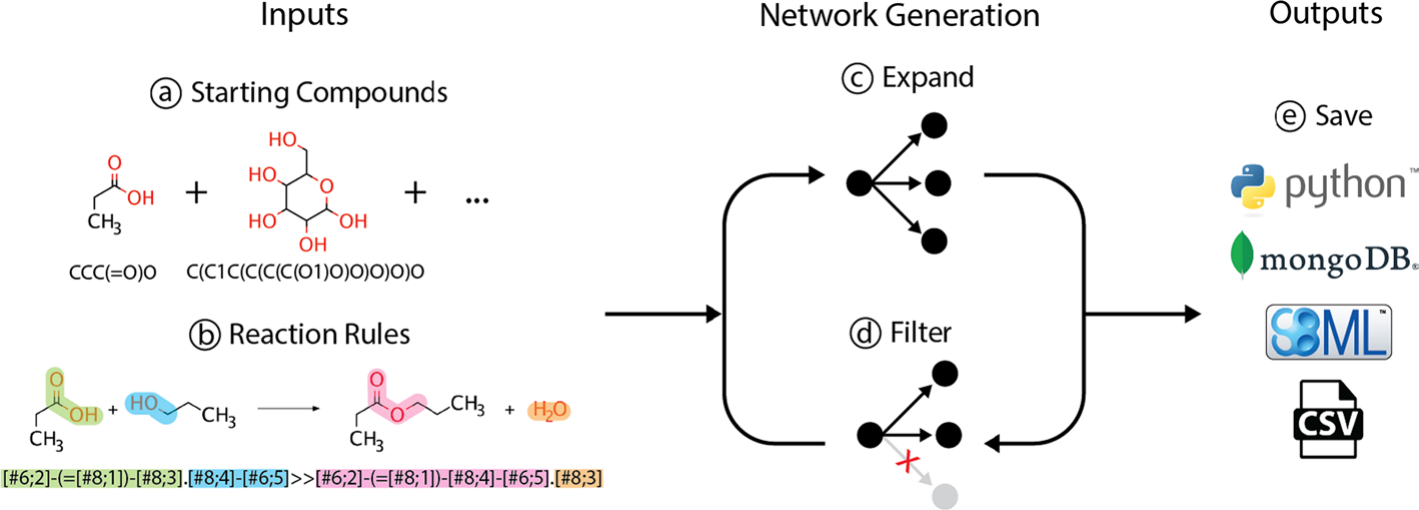
Overview of Pickaxe. First, **a** starting compounds represented by SMILES and **b** reaction rules represented as SMARTS are loaded. Next, the **c** reaction rules and **d** filters are applied iteratively for a specified number of generations. Finally, **e** the resulting network is saved in one of three formats or used further in the python script

At its core, the most basic network expansion with Pickaxe consists of the following: user-specified compounds, reaction rules, iterative network generation, and data output for further analysis (Fig. 1). Pickaxe comes packaged with a default set of reaction rules and multiple features, but it is possible for user-defined reaction rules to be created and utilized [20]. Additionally, filters can be used to help guide network generation by removing specific compounds and reactions on-the-fly. After a Pickaxe object generates a network, the results can be saved to MongoDB, a csv file, serialized with pickle, or used directly within a python script.

### Starting compounds

A file containing the structures of the starting compounds is required for each Pickaxe run (Fig. 1a). Compounds are represented as SMILES, a string based representation of compound structure, and are loaded into the model via a CSV file consisting of two columns, an id column, and a SMILES column [30]. Once loaded into the Pickaxe object, each compound is neutralized, and any stereochemistry is removed by default. The rules provided with Pickaxe do not utilize stereochemistry, but it is possible to retain the stereochemical information when loading compounds if user-defined rules contain stereochemical information.

### Reaction rules

**Fig. 2:**
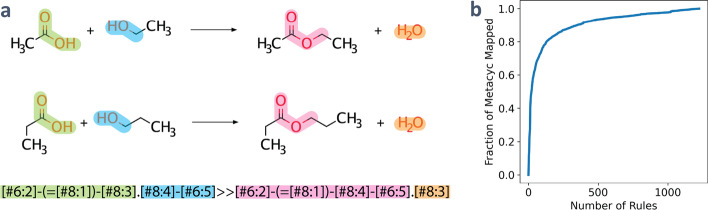
Generation and selection of rules. **a** Rules are generated by identifying common patterns within reactions and translating those into a reaction SMARTS representation. **b** A plot of the cumulative distribution function for the JN1224min rules showing the fraction of MetaCyc mapped for a specific number of rules used

Pickaxe utilizes reaction rules that are written in SMARTS (Fig. 2a), which are strings that describe a reaction by specifying a substructure that is capable of mapping onto reactants and transforming them to generate products. Pickaxe utilizes SMARTS written according to the daylight specifications [30]. Users can write and use their own set of SMARTS, or use the ruleset included with Pickaxe. The provided rules, known as JN1224min, consist of 1224 generalized rules that represent a minimal, yet comprehensive, ruleset that maps MetaCyc reactions [20]. The JN1224min ruleset also includes a list of UniProt IDs for each rule corresponding to enzymes that are associated with the MetaCyc reactions used to derive the rules, providing a list of potential enzymes for a predicted reaction. It is important to note that each rule maps a different fraction of MetaCyc reactions and when using a fraction of the rules, the rules are applied in the order of most mapped reactions to the fewest. As a result, only a fraction of the rules to map a majority of the MetaCyc reactions (Fig. 2b). For example, 353 rules map 90% of the Metacyc reactions, while it takes approximately double that number of rules, 658, to map 95%. Using every rule will enable the most comprehensive expansion, but also may lead to computationally intractable networks depending on the number of rules, starting compounds, and generations used. To give the user control over this tradeoff, it is possible to utilize built-in filters or to specify a subset of the rules to be used by either specifying the fraction of MetaCyc covered or the number of rules to use (in order of increasing MetaCyc coverage). Additionally, rules may be selected or excluded based on features such as oxygen-dependence, aromaticity, and functional groups.

### Network expansion

Once the input compounds and the reaction rules are defined, the RDKit, a cheminformatics program, is used to apply the SMARTS rules to carry out the transformation of reactants into products [31]. These rules are applied iteratively for a specified number of generations (Fig. 1c), and resulting reactions are checked to ensure all compound structures are valid and all reactions are atom balanced. The reaction generation yields new compound and reaction objects, which are stored in dictionaries in the Pickaxe object and can be directly accessed. Every compound in the network is assigned a unique ID generated by taking a hash of the compound’s InChiKey which ensures consistency between Pickaxe runs. Compound objects within Pickaxe also contain information about the structure of the compound, which reactions they participate in, and the first generation in which they were seen. Reaction objects contain information about which compounds participate, their stoichiometry, and the reaction operator that maps that reaction. A unique ID is assigned to each reaction which is generated by taking a hash of the reaction formula.

### Filters

Depending on the number of compounds and rules used, networks can quickly become large due to the combinatorial nature of network generation, potentially complicating analysis or rendering the expansion computationally intractable. To combat this, Pickaxe provides the ability to apply filters during the network generation process (Fig. 1d). After the expansion of each generation, Pickaxe will apply user-specified filters, which flag reactions and compounds and result in the removal of them from the network. Multiple filters can be applied simultaneously, giving users the options to filter both compounds and reactions in the same run. There are three types of filters provided with Pickaxe: those which examine individual compound properties, those that compare compounds to a set of provided targets, and those that look at properties of reactions. Custom, user-defined filters may also be defined and integrated into a Pickaxe run.

### Compound property filters

The two simplest filters provided with Pickaxe utilize the molecular weight (MW) and the atomic composition of an individual compound to determine if it should be removed from the network.

The molecular weight filter accepts a MW range that a compound must exist in if it is to be considered for further expansion. Similarly, the atomic composition filter specifies a range for each element in the molecule, allowing for the restriction of molecules to contain only specific compositions.

### Similarity filters

When generating networks in which specific targets are sought, it is useful to also investigate if a compound is likely to produce a target. To accomplish this, Pickaxe uses chemical similarity to determine how similar a compound is to the targets and uses that value to aid in filtering out reactions which contain dissimilar molecules. Pickaxe calculates similarity by generating chemical fingerprints using the RDKit fingerprint, a bit vector which represents the features of the compound, for two compounds and calculating the Tanimoto similarity score between these two fingerprints [32]. This process is repeated for each compound of the current generation to calculate the maximum similarity score of that compound with each of the targets, resulting in a distribution of similarity scores. This distribution is then used in two different ways: a similarity cutoff filter and a similarity sampling filter. The cutoff filter simply removes any compounds whose maximum similarity score is below a specified threshold. The sampling filter randomly selects a specified number of compounds to keep from a weighted Tanimoto distribution defined by the user.

### Metabolomics filter

In order to limit predicted compounds to only those that annotate experimental metabolomics data, the metabolomics filter can be used. It takes as input a list of experimental mass-to-charge (m/z) values and a list of possible adducts. These adducts are used to calculate the possible m/z values for each predicted compound, and compounds that can be combined with one of these specified adducts to have one of the input m/z values (within some user-defined tolerance) are kept for expansion. This allows Pickaxe expansions to be efficiently constrained by experimental metabolomics data, if available.

### Thermodynamics filter

Pickaxe supports the calculation of the Gibbs free energy of reaction in the aqueous phase for reactions within the generated network and provides a thermodynamics-based filter to determine reaction feasibility. eQuilibrator, a biochemical thermodynamic calculator, was integrated into Pickaxe allowing for the calculation of three separate Gibbs free energies: (1) the standard Gibbs free energy of reaction, (2) the physiological Gibbs free energy of reaction, where ionic strength is 0.25 M, pH is 7.5, and all concentrations are 1 mM, and (3) the adjusted Gibbs free energy of reaction, where the ionic strength and pH are specified by the user [33]. With these Gibbs free energies, the thermodynamics filter can remove reactions by specifying a minimum and maximum Gibbs free energy of reaction. While the utilization of the Gibbs free energy of reaction for an individual reaction provides a metric for feasibility, when considering pathways, it may be important to consider the interplay between each reaction. One method to accomplish this is to calculate the Max–min Driving Force (MDF), which identifies the thermodynamic bottleneck for a given pathway and has been used to determine the feasibility of biosynthetic pathways [34]. Pickaxe does not calculate this value directly but generates the eQuilibrator objects necessary to easily interface with the equilibrator-api python package allowing for more sophisticated thermodynamics analysis.

### Feasibility filter

The reaction rules used by Pickaxe are defined by generalized transformation rules and are capable of generating reactions that are not experimentally feasible. To identify and exclude these reactions from any potential pathways, Kim et al. developed a machine learning approached entitled Deep learning-based Reaction Feasibility Checker (DeepRFC) [35]. DeepRFC utilizes substrate-product pairs within KEGG reactions to train a deep neural net to predict a reaction’s feasibility. This software has been incorporated into Pickaxe, allowing for efficient filtering of reactions likely to be infeasible from the network on-the-fly.

### Outputs

Pickaxe generated networks can be stored and accessed in three different formats, with each format allowing for access to information on the compounds, reactions, reaction rules, and target molecules (Fig. 1e). Most directly, the generated Pickaxe object provides a method to serialize itself utilizing the Pickle module and to subsequently generate a new Pickaxe object from the serialized file. Pickaxe offers MongoDB support to read and write the results in a JSON format from a MongoDB database. The generated database consists of collections of compounds, reaction, targets (if specified), and operators. Pickaxe also supports saving the results as a Systems Biology Markup Language (SBML). File, a standard file format that allows Pickaxe to be exported and used for incorporation into other tools [36]. Lastly, the results can be stored in CSV files that contain compound, reaction, operator, and target information.

## Results and discussion

Pickaxe provides a framework to predict promiscuous reactions and generate reaction networks for a variety of applications. To highlight the flexible nature of Pickaxe, example expansions were created which demonstrate Pickaxe’s ability for novel pathway generation for metabolomics and biosynthetic pathway design. These runs provide context for rule and filter selection for tailoring network generation towards a specific application. For all runs, one of two computational set ups were used to run the examples: a node on a supercomputing cluster using 50 Intel® Xeon® Gold 6230 cores or a laptop containing a 2.2 GHz 6-Core Intel Core i7 processor.

### Expansion of common biological databases

Pickaxe was originally developed to generate networks for use in untargeted metabolomics [19]. Given a series of unannotated metabolomics peaks, it is possible to utilize the promiscuous reaction operators to extend a single generation from common biological databases. The resulting network is then checked for matches with the unannotated peaks, providing prospective compounds and reactions that help to elucidate metabolism for an organism. It is important to create an exhaustive network to identify as many potential compound peak pairs as possible. To highlight the size of networks achievable, three commonly used biological databases were selected and expanded: the Kyoto Encyclopedia of Genes and Genomes (KEGG) (Release 99.0), EcoCyc (Version 25.1), and the Yeast Metabolome Database (YMDB) (Version 2.0) [37–39]. Each database was filtered to only contain compounds whose molecular weights are less than 600 g/mol. The top 500 rules (94% of MetaCyc mapped) from the JN1224min ruleset were applied to the starting compounds for one generation. For each database, approximately two orders of magnitude more compounds are generated in comparison to the starting compounds, and a similar number of reactions are generated, highlighting the ability of Pickaxe to generate a large number of predicted compounds and reactions (Table 1).

**Table 1:** Single generation expansion of biological databases

Database	Starting compounds	Generated compounds^a^	Generated reactions^b^
KEGG	12,731	9,562,940	13,759,651
EcoCyc	1,923	906,086	1,388,653
YMDB	1,035	575,262	892,343

^a^Unique compounds produced after one expansion

^b^Unique reactions connecting a starting compound to a generated compound

### Rate of network expansions

**Fig. 3:**
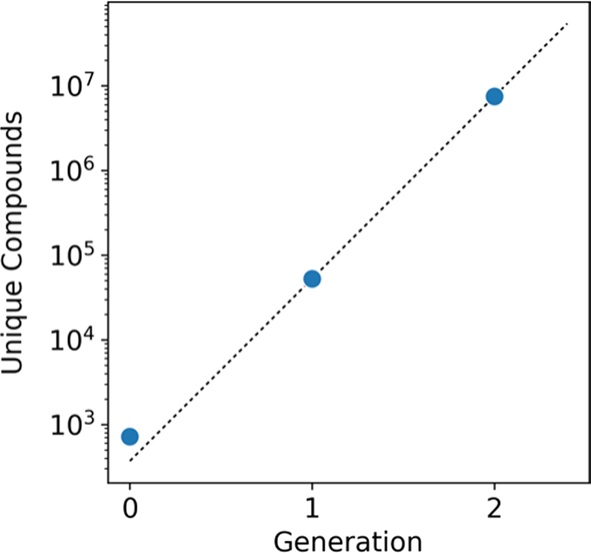
A Two generation expansion and insertion into a mongo database of a reduced YMDB using 100 rules results in an exponentially increasing network size

While an exhaustive network is useful for certain applications, network sizes increase exponentially as the number of generations increases (Fig. 3a). Depending on computational resources, it can become computationally intractable to generate a network as the number of rules, starting compounds, and generations used increases. To highlight this behavior, compounds less than 300 g/mol molecular weight taken from YMDB were expanded using the top 100 rules (76% MetaCyc coverage). With each generation, the number of unique compounds produced increased exponentially, resulting in a total of two generations and 12 million new compounds being generated and inserted into a mongo database in approximately nine hours. An attempt to create a third generation was allowed to run for a week but was not completed within that time. Depending on the expansion parameters and the computational resources available it may not be possible to create the desired network for an application due to time or memory constraints (Additional File 1). By varying the number of rules, specifying targets, and applying filters it is possible to design expansions for a variety of applications on a variety of computational resources (Fig. 3b).

### Pruning networks with a similarity cutoff filter

The similarity cutoff filter provides a method to control the generated network size by removing compounds that are not similar enough to a set of target compounds from the network. Before each expansion, the similarity score between a compound and each of the target compounds is calculated and if the compound’s maximum similarity score is below a specified threshold it is removed from the network. By varying this similarity cutoff, it is possible to exert control over the size of the generated network to best suit the computational resources available while still producing a network containing target compounds. To demonstrate this filter, compounds less than 600 g/mol from YMDB were expanded using the top 353 JN1224 rules (90% MetaCyc mapped) with similarity cutoffs of 0, 0.2, 0.4, 0.6, and 0.8. A set of 250 target compounds was selected by randomly choosing compounds from the KEGG database containing between four and nine carbons that are not also within YMDB. Additionally, an atomic composition filter was used to limit the number of carbon atoms contained in non-cofactor molecules to fewer than twelve. All runs were allowed to run for one week.

**Fig. 4:**
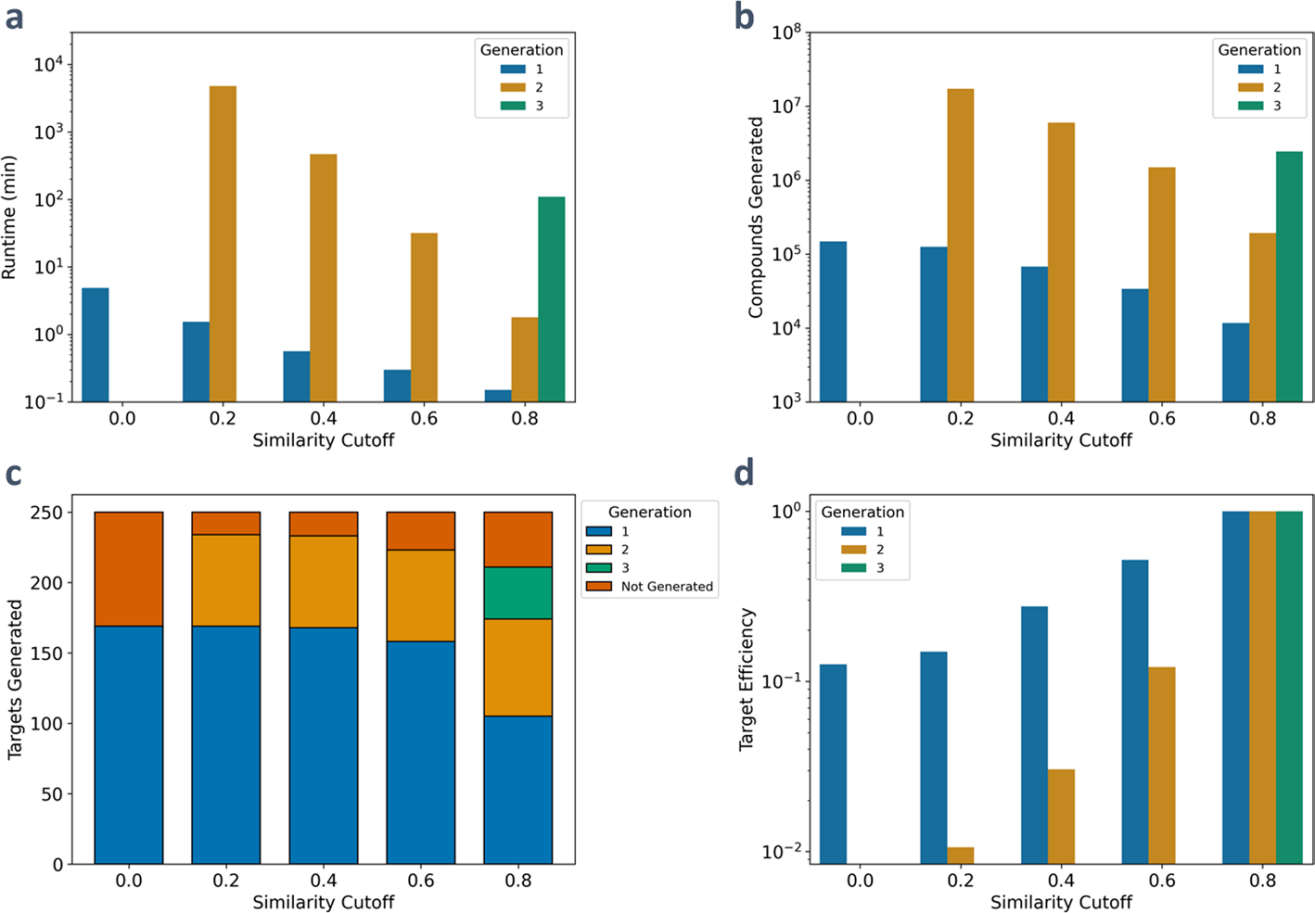
Results of a Tanimoto cutoff expansion of YMDB compounds less than 600 g/mol using 353 JN1224 rules. A set of 250 KEGG compounds containing between four and nine carbon atoms not in the YMDB database were selected as targets. **a** The runtime for each generation for the different cutoffs. **b** Total number of compounds generated for varying similarity cutoffs. **c** The total number of targets generated for each similarity cutoff. **d** The efficiency of target generation for each similarity cutoff

When expanding without a filter, only one generation was able to be generated before the program timed out (Fig. 4a). By increasing the cutoff, it was possible to produce two generations in 80, 8, and 0.5 h for the 0.2, 0.4, and 0.6 thresholds, respectively. Using an even more strict cutoff of 0.8 enabled three generations to be produced in 2 h. The reduction in run time is associated with the decrease in total compounds and reactions produced (Fig. 4b). However, this increase in the threshold has a tradeoff, as resulting networks contain fewer compounds and reactions and therefore potentially decreases the number of targets reached. For this system in particular, the total number of targets produced does not significantly decrease until the 0.8 cutoff (Fig. 4c). Although the number of targets reached decreases with increasing cutoff, the efficiency in creating these targets improves. Defining the efficiency of target generation for a cutoff, $i$, at generation, $j$, compared to a cutoff of 0.8 at generation, *j*, as

1 $$Target Efficiency=\frac{\left(\frac{Target{s}_{i,j}}{Compound{s}_{i,j}}\right)}{\left(\frac{Target{s}_{0.8,j}}{Compound{s}_{0.8,j}}\right)},$$

it is possible to compare each expansion’s ability to produce the least number of compounds for each target (Fig. 4d). As the similarity cutoff increases, this efficiency increases as well, indicating that despite smaller networks being produced, relatively more targets are being generated. For example, a cutoff of 0.4 yielded a similar number of targets generated as no cutoff but produced approximately three times fewer compounds. By tailoring the similarity cutoff, this filter provides a powerful tool to adapt network generation both for specific applications as well as computational resource considerations.

### Generating targets with a similarity sampling filter

The similarity sampling filter utilizes a weighted distribution of the similarity scores calculated with a set of target compounds to sample a specified number of random compounds to be expanded. By controlling the number of compounds generated and the weighted distribution, it is possible to exert control over both the size and the similarity score distribution of the resultant network. To demonstrate this filter, compounds with molecular weight less than 600 g/mol from YMDB were expanded using the top 353 JN1224min rules (90% MetaCyc mapped). Three networks were generated by specifying the sampling filter to select 2000 compounds from distributions generated from scaling the similarity score distribution to the zeroth, third, and sixth powers. The same input compounds, targets, and computational parameters were used as the similarity threshold filter.

**Fig. 5:**
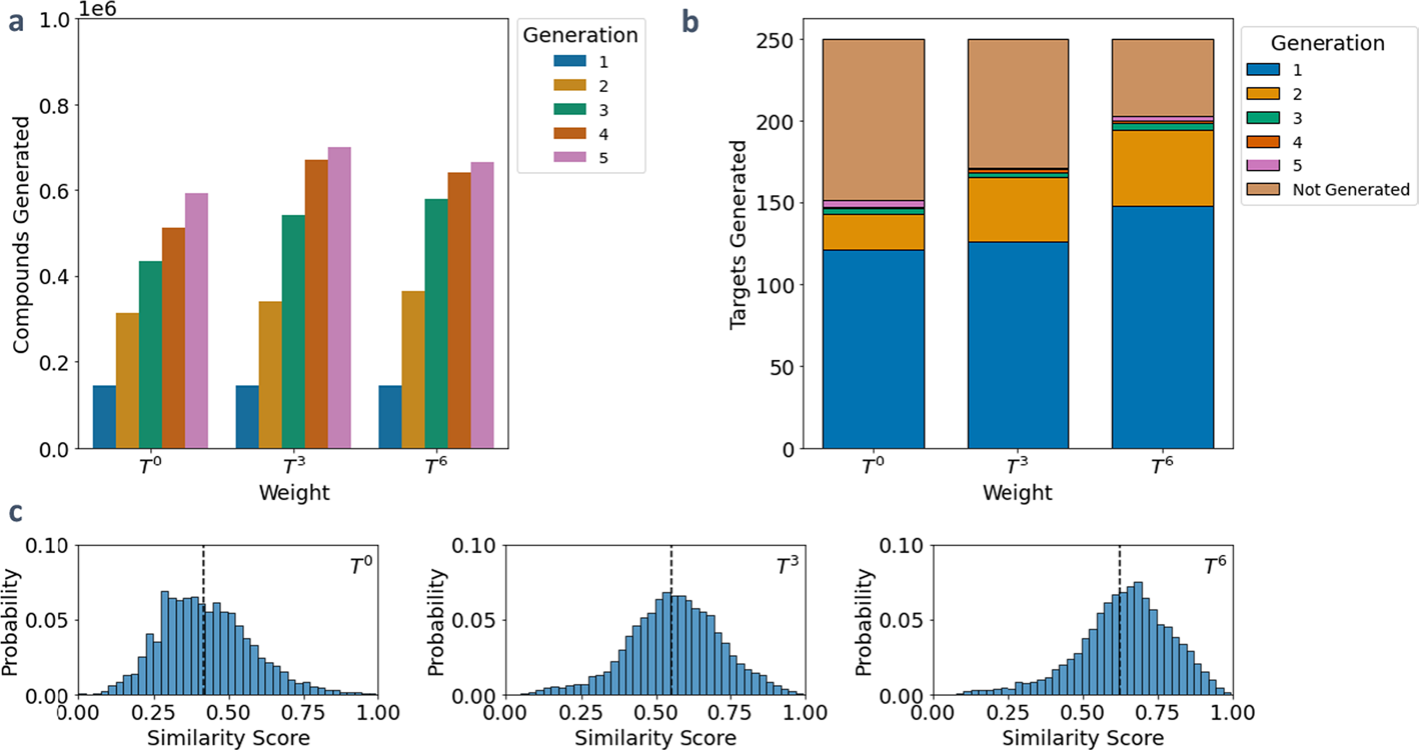
Results of a similarity sampling expansion of YMDB compounds less than 600 g/mol using 353 JN1224 rules. A set of 250 KEGG compounds containing between four and nine carbon atoms not in the YMDB database were selected as targets. **a** The total number of compounds produced per generation. Before each generation, 2000 compounds were sampled to be reacted. **b** The total number of targets produced per generation and **c** the similarity distribution of each resultant network. This distribution consists of the maximum similarity value of each network compound to the targets. The vertical line signifies the mean similarity score

Unlike expansions with the similarity cutoff filter or with no filter, the similarity sampling filter selects only a fixed number of compounds to react, resulting in network sizes that do not grow exponentially (Fig. 5a). This filter enables the production of a large number of generations, allowing for longer pathways to targets to be produced. In this example, each weighting function generates the most targets in the first generation, and then each subsequent generation reduces the number of targets generated per generation. Weighting the similarity score higher results in the most target compounds generated, as there is a higher probability of selecting compounds that are more similar to the targets to expand (Fig. 5b). However, when weighting with a higher power, the similarity distribution reveals that the compounds in the network are more likely to be similar to the targets, while a uniform sampling yields a broader network with more variety in chemical similarity (Fig. 5c). Altering the number of compounds sampled and the sampling weighting functions provides a flexible method to explore the expansion with control over the chemical space explored and the similarity of the resultant network to the target set.

### Generating candidates for metabolomics with non-similarity filters

In addition to filtering compounds based on chemical similarity to a set of targets, Pickaxe includes three additional filters utilizing either metabolomics data, chemical reaction feasibility, or reaction thermodynamics to remove compounds and reactions on-the-fly. To highlight the use of the three non-similarity filters an expansion was created to identify potential matches in an unannotated *E. coli* metabolomics dataset using the EcoCyc database. Additionally, this example was done on a personal computer, demonstrating Pickaxe’s utility even without access to a super computer. For this application, the ideal generated network will provide a network containing prospective annotations for the experimentally identified peaks while also containing reactions that are most likely to happen. Through the application of these three filters, it is possible to achieve these goals, demonstrating the built-in filters included with Pickaxe as well as the flexibility of utilizing multiple filters.

Compounds less than 600 Da selected from the EcoCyc database were expanded for two generations using the top 50 JN1224min rules (65% MetaCyc mapping). 200 unannotated peaks with m/z values between 150 and 300 Da were selected from an experimental dataset as inputs to the metabolomics filter [20, 40]. Multiple expansions were generated using combinations of the metabolomics, feasibility, and thermodynamic filters to remove reactions on-the-fly between generations. The thermodynamics filter was set to remove reactions with a physiological Gibbs free energy greater than 50 kJ/mol. It is important to note that it is possible that eQuilibrator does not return a Gibbs free energy for a reaction, as the reaction falls outside the scope of its predictions. These reactions were left to remain in the network, as the thermodynamics filter cannot comment on their feasibility. Lastly, an atomic composition filter was used to limit the number of carbon atoms of non-cofactor compounds to fewer than twelve.

**Fig. 6:**
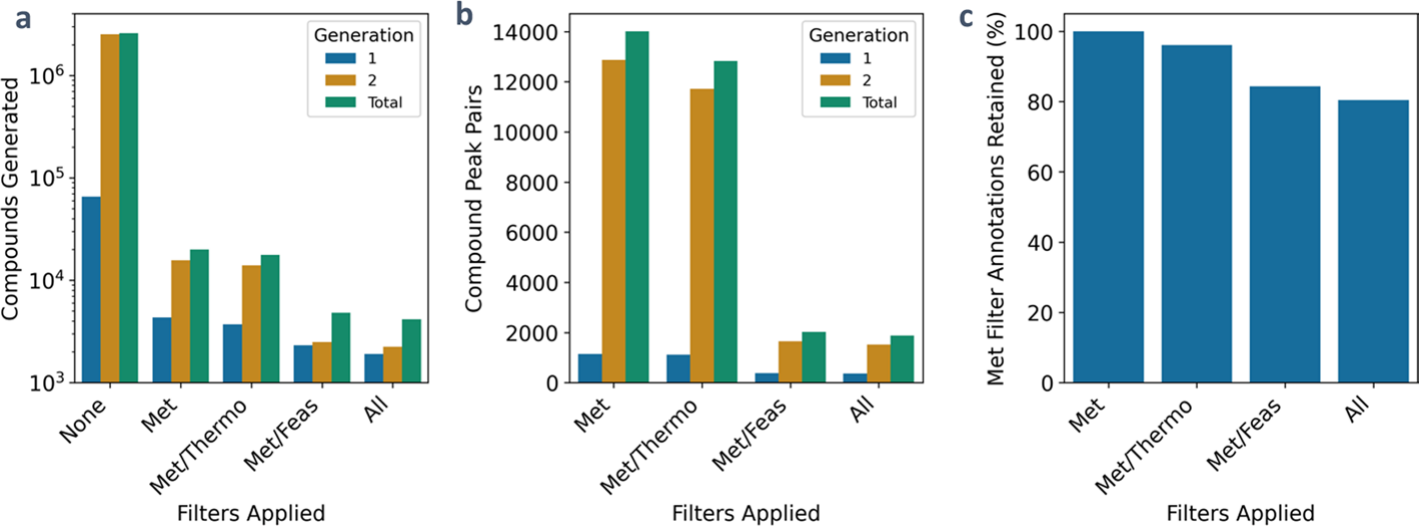
Annotation of an untargeted *E. coli* metabolomics dataset. Compounds less than 600 Da selected from the EcoCyc database were expanded using the top 50 JN1224min rules utilizing metabolomics, thermodynamic, and feasibility filters. **a** Number of unique compounds produced for each generation for a mixture of filters, **b** the total number of matches between compounds and unannotated peaks, and **c** the percentage of metabolic filter only annotated peaks retained (still matches at least one candidate compound based on m/z) for each set of filters

Without any filters, approximately 2.5 million total compounds were produced in two generations (Fig. 6a). Repeating this expansion with the metabolomics filter, which only keeps compounds whose m/z value matches one of those in the unannotated metabolomics dataset, greatly reduced the size of this network by two orders of magnitude. The resulting metabolomics filter network contains 19,967 unique compound peak pairs matching 51 peaks (Fig. 6a, b). While the network generated using the metabolic filter can be used to identify reactions and compounds to annotate this data, it is possible to utilize multiple filters to further reduce the generated network by removing undesired compounds and reactions.

Because of their generalized nature, promiscuous reaction rules may create reactions that do not occur in nature. To remove these infeasible reactions, the chemical feasibility and thermodynamic feasibility of these reactions were determined and used to reduce the network size. The combination of thermodynamics and metabolomics filters does not significantly affect either the number of compounds or compound peak pairs when compared to the metabolomics filter alone (Fig. 6a, b). When the feasibility filter is combined with the metabolomics filter, however, the number of both the compounds and compound peak pairs is decreased by an order of magnitude. Utilizing all three filters at once yields a similar network to that of the feasibility and metabolomics filter combination. Regardless of the filter combination used, the total number of peak annotations generated is very similar for each filter combination, showing that even though the network size was greatly reduced through filters, a breadth of compounds was still produced that match the unannotated peaks (Fig. 6c). Through the application of multiple filters to guide the network expansion, it is possible to easily tailor a given expansion to match the needs of a given application.

## Conclusions

Pickaxe is a user-friendly, open-source, customizable network generation tool that allows for the creation of novel compounds and reactions through reaction rules. A network expansion requires the specification of starting compounds and reaction rules, with the option to add filters that enable on-the-fly filtering of a generated network. Pickaxe comes prepackaged with JN1224min, a set of enzymatically derived reaction rules generated from MetaCyc reactions, and also supports the definition of user-defined rules. During an expansion, it is possible to apply filters that reduce the network by filtering out compounds and reactions using a combination of thermodynamic, metabolomic, feasibility, or similarity filters. Generated networks can be output in multiple formats, allowing for easy storage of results for later analysis. Through its customizable inputs and documentation, Pickaxe provides a user-friendly method to generate, filter, and store runs that are tailor-fit for given applications.

## Availability and requirements

Project name: Pickaxe

Project home page: Code documentation and tutorials can be found at https://mine-database.readthedocs.io/en/latest/

Code availability: Pickaxe is available as a pypi package at https://pypi.org/project/minedatabase/ and the source code is available at https://github.com/tyo-nu/MINE-Database

Operating System(s): Platform independent

Programming language: Python

Other Requirements: Python 3.7 or higher. If saving results using MongoDB then a mongo server must be installed locally or remotely on a server.

License: MIT.

Any restrictions to use by non-academics: No restrictions.

## Supplementary Information

**Additional file 1.** Pickaxe runtime performance benchmarks.

## Data Availability

All raw data used in this study are published and available for download [37–40]. Code to generate the data used for analysis and run the analysis can be found at https://github.com/tyo-nu/pickaxe_paper. Generated networks are available from the corresponding author on reasonable request.

## Abbreviations

CSV: Comma separated values
KEGG: Kyoto encyclopedia of genes and genomes
YMDB: Yeast Metabolome Database
