# Supplementary material for: Pickaxe: a Python library for the prediction of novel metabolic reactions
Source: BMC Bioinformatics. 2023 Mar 22;24:106. doi: 10.1186/s12859-023-05149-8 (PMC10031857; doi:10.1186/s12859-023-05149-8)
Supplement: Supplementary file 1 — Additional file 1. Pickaxe runtime performance benchmarks. [file 12859_2023_5149_MOESM1_ESM.docx]

**Pickaxe: a Python library for the prediction of novel metabolic reactions**

Kevin M. Shebek^1,2,3^, Jonathan Strutz^1,2,3^, Linda J. Broadbelt^1,2^, Keith E.J. Tyo^1,2,3*^

**^1^Department of Chemical and Biological Engineering, Northwestern University, Evanston, IL, USA 60208**

**^2^Center for Synthetic Biology, Northwestern University, Evanston IL, USA 60208**

**^3^Chemistry of Life Processes Institute, Northwestern University, Evanston, IL, USA 60208**

*Correspondence: [k-tyo@northwestern.edu](mailto:k-tyo@northwestern.edu)

**Supplemental Information**

**Pickaxe Performance Benchmarking**

**a**

**b**


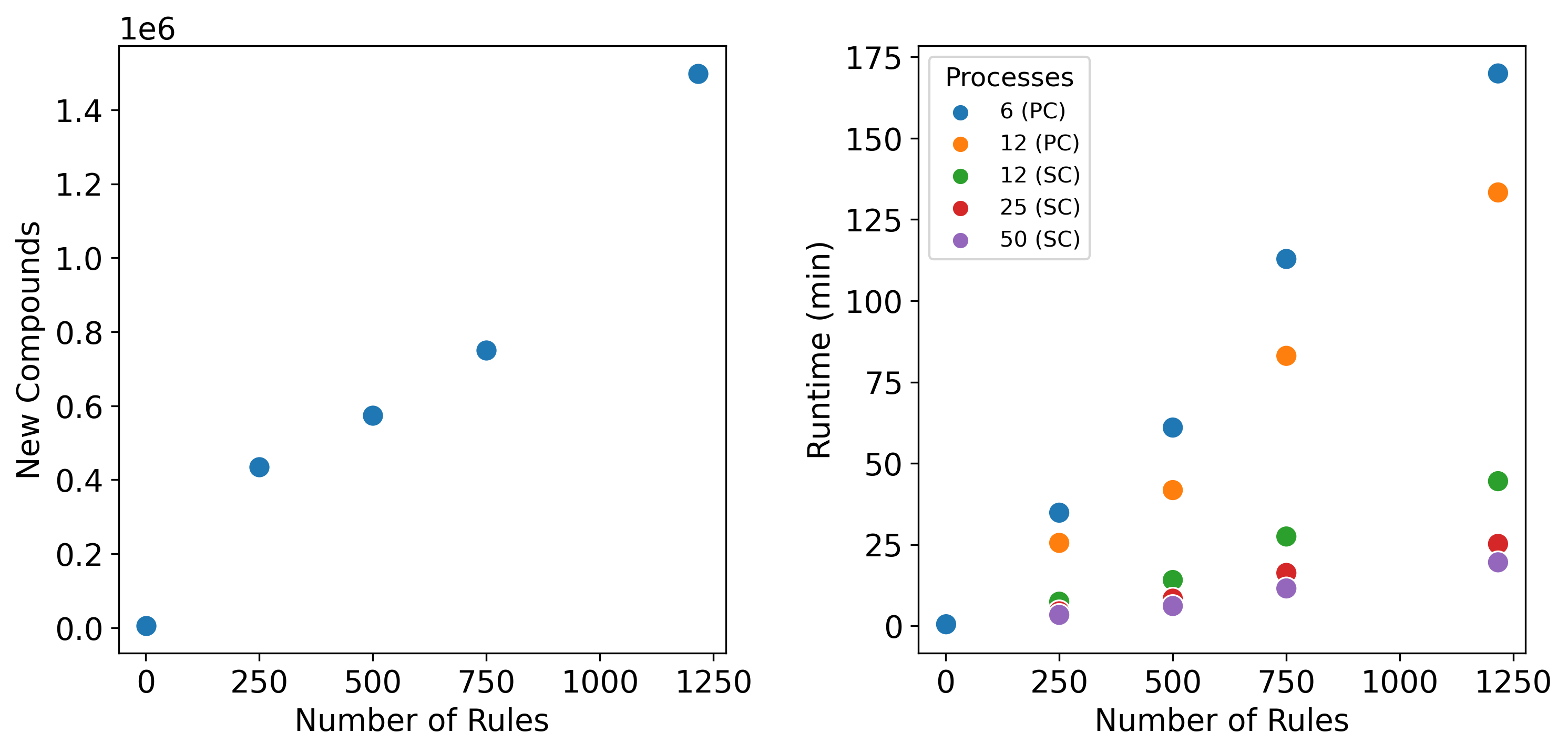


**Figure S1.** (a) An increasing number of reaction rules (1, 250, 500, 750, and 1224) applied to the yeast metabolome database produces more rules. However, for this expansion the rate of new compound production is not linear with respect to the number of rules used. (b) Due to this nonlinearity in the increase in number of compounds generated, the runtime also does not increase linearly. The runtime is also dependent on the number of cores and the computer. With fewer cores the runtime increases, and the runtime is quicker on a supercomputer (SC) with better processors than a personal computer (PC).

**Table S1. Benchmark results of a yeast metabolome database expansion**

| Processes | Computer | Number of Rules | Fraction of MetaCyc Mapped | Run Time (min) | New Compounds | New Reactions |
| --- | --- | --- | --- | --- | --- | --- |
| 6 | PC | 1 | 0.05 | 28.55 | 5719 | 5634 |
| 6 | PC | 250 | 0.84 | 2088.60 | 434513 | 601004 |
| 6 | PC | 500 | 0.92 | 3659.82 | 574243 | 892343 |
| 6 | PC | 750 | 0.96 | 6771.93 | 750642 | 1254441 |
| 6 | PC | 1216 | 1.00 | 10196.52 | 1498567 | 2082771 |
| 12 | PC | 1 | 0.05 | 12.11 | 5719 | 5634 |
| 12 | PC | 250 | 0.84 | 1531.16 | 434513 | 601004 |
| 12 | PC | 500 | 0.92 | 2505.25 | 574243 | 892343 |
| 12 | PC | 750 | 0.96 | 4983.43 | 750642 | 1254441 |
| 12 | PC | 1216 | 1.00 | 7999.79 | 1498567 | 2082771 |
| 12 | SC | 1 | 0.05 | 5.52 | 5719 | 5634 |
| 12 | SC | 250 | 0.84 | 445.33 | 434417 | 600905 |
| 12 | SC | 500 | 0.92 | 845.05 | 574105 | 892191 |
| 12 | SC | 750 | 0.96 | 1648.66 | 750469 | 1254228 |
| 12 | SC | 1216 | 1.00 | 2670.07 | 1497840 | 2082001 |
| 25 | SC | 1 | 0.05 | 7.20 | 5719 | 5634 |
| 25 | SC | 250 | 0.84 | 266.89 | 434417 | 600905 |
| 25 | SC | 500 | 0.92 | 501.00 | 574105 | 892191 |
| 25 | SC | 750 | 0.96 | 976.07 | 750469 | 1254228 |
| 25 | SC | 1216 | 1.00 | 1512.35 | 1497840 | 2082001 |
| 50 | SC | 1 | 0.05 | 4.30 | 5719 | 5634 |
| 50 | SC | 250 | 0.84 | 201.80 | 434417 | 600905 |
| 50 | SC | 500 | 0.92 | 365.50 | 574105 | 892191 |
| 50 | SC | 750 | 0.96 | 690.40 | 750469 | 1254228 |
| 50 | SC | 1216 | 1.00 | 1172.09 | 1497840 | 2082001 |
